# Multi-Laboratory Evaluation of Prototype Dried Blood Spot Quality Control Materials for Creatine Kinase-MM Newborn Screening Assays

**DOI:** 10.3390/ijns9010013

**Published:** 2023-02-28

**Authors:** Paul Dantonio, Norma P. Tavakoli, Brooke Migliore, Elizabeth McCown, Timothy Lim, Sunju Park, Michele Caggana, Katerina S. Kucera, Han Phan, Natalie Street, Konstantinos Petritis, Robert F. Vogt

**Affiliations:** 1 Newborn Screening and Molecular Biology Branch, Division of Laboratory Sciences, National Center for Environmental Health, Centers for Disease Control and Prevention, Atlanta, GA 30333, USA; 2 Wadsworth Center, Division of Genetics, New York State Department of Health, Albany, NY 12208, USA; 3 RTI International, Research Triangle Park, Durham, NC 27709, USA; 4 Department of Pediatrics, Heersink School of Medicine, University of Alabama at Birmingham, Birmingham, AL 35233, USA; 5 Rare Disease Research, Atlanta, GA 30329, USA; 6 Birth Monitoring and Research Branch, Division of Birth Defects and Infant Disorders, National Center on Birth Defects & Developmental Disabilities, Centers for Disease Control and Prevention, Atlanta, GA 30333, USA

**Keywords:** Duchenne muscular dystrophy, newborn screening, evaluation, specimen quality, dried blood spots

## Abstract

Pilot studies to detect newborns with Duchenne Muscular Dystrophy (DMD) by newborn bloodspot screening (NBS) have been conducted under the New York State Newborn Screening Program (NYS) and are currently in progress as part of the Early Check Program at Research Triangle Institute (RTI) International. The Newborn Screening Quality Assurance Program (NSQAP) at the U.S. Centers for Disease Control and Prevention (CDC) produced a set of seven prototype dried blood spot (DBS) reference materials spiked with varying levels of creatine kinase MM isoform (CK-MM). These DBS were evaluated over a 3-week period by CDC, NYS, and RTI, all using the same CK-MM isoform-specific fluoroimmunoassay. Results from each laboratory were highly correlated with the relative proportion of CK-MM added to each of the six spiked pools. Based on reference ranges established by NYS and RTI for their pilot studies, these contrived DBS collectively spanned the CK-MM ranges found in typical newborns and the elevated ranges associated with DMD. This set allows quality assessment over the wide range of fluctuating CK-MM levels in typical and DMD-affected newborns.

## 1. Introduction

Duchenne Muscular Dystrophy (DMD) is the most prevalent of the dystrophinopathies, muscle disorders caused by pathogenic variants of the *dystrophin* gene [1]. DMD can be detected in affected newborns by elevated blood levels of creatine kinase (CK), and it has long been considered a potential target condition for newborn bloodspot screening (NBS) [2,3]. Recent advances in gene therapies for DMD have renewed interest in NBS to improve early detection and timing of optimal intervention [4]. Along with these advances in treatment, a fluoroimmunometric assay specific for the MM isoform of CK has recently been developed using instrumentation designed for NBS; it received authorization by the U.S. Food and Drug Administration in December 2019 [5,6]. This assay has been used in several recent DMD-NBS pilot studies [4,6,7,8,9,10,11,12]. Globally, at least one newborn screening program has included DMD in its routine NBS panel using this assay [13], and DMD has recently been nominated for inclusion in the U.S. Recommended Uniform Screening Panel (RUSP).

NBS laboratories are required to follow a quality assurance (QA) strategy determined by the laboratory, local guidelines, and accreditation organizations. As part of QA, laboratories perform proficiency testing (external quality assessment), and they monitor performance using quality control (QC) materials [14]. External QC materials (specifically dried blood spot quality control materials (DBS-QC)) are used to supplement the method or kit controls, and when assayed periodically over time, they facilitate the assessment of long-term stability of methods. The importance of external QC materials cannot be overstated, as evidenced by the cessation in 2011 of the 21-year DMD-NBS program in Wales, primarily due to the lack of such external QC materials [15].

The Newborn Screening Quality Assurance Program (NSQAP) at the U.S. Centers for Disease Control and Prevention (CDC) produces and distributes DBS-QC for use as external reference materials by NBS laboratories [16]. In collaboration with laboratories at the New York State Department of Health (NYS) and RTI International (RTI), we report here the first multi-laboratory evaluation of contrived DBS spiked with varying CK-MM levels selected to span the range of typical and DMD-affected newborns and measured by the isoform-specific fluoroimmunometric assay.

## 2. Materials and Methods

### 2.1. Preparation of DBS-QC

The prototype DBS materials comprised 7 different levels of CK-MM ranging from a base pool with negligible CK-MM activity (Pool A) to a highest-level pool (Pool G). Pool A was prepared from leuko-depleted red cells (BioIVT, Hicksville, NY, USA) which were washed three times with normal saline then reconstituted to a 50% hematocrit with charcoal-stripped serum (SeraCare Life Sciences Inc, Milford, MA, USA; catalog number 1800-0006) heat-inactivated at 56 °C for four hours. Pool G was made by adding rehydrated CK-MM enzyme (Sigma-Aldrich, St. Louis, MO, USA, catalog number C9858, lot SLBM5232V) to Pool A to achieve a calculated target level of 5000 ng/mL based on specific activity information provided with the CK-MM. Pools B through F were admixtures of Pool A and Pool G (Table 1) [16,17]. 

The pools were dispensed onto filter paper cards cleared for use as a blood spot collection device (grade 903, lot number 7105618 W171, Whatman, Maidstone, UK). For each pool, a card printed with 15 outlined circles was manually spotted with 100 uL per circle of the same pool. The cards were allowed to dry overnight under ambient conditions and then packaged in low-permeability ziplock bags (Thermo Fisher Scientific, Inc., Waltham, MA, USA, catalog number 19240127) containing desiccant packs (Desiccare, Inc., Richland, MS, USA, catalog number 01AD11A12). Complete sets including one card each of Pools A through G were sent overnight at ambient temperature to NYS and RTI, and one set was retained at CDC. The packaged cards were stored at −20 °C between analyses. For analysis, bagged cards were removed from the freezer and allowed to reach room temperature before removal from the desiccated bag.

### 2.2. Study Protocol and Data Analysis

Each of the three laboratories conducted weekly runs over the same three-week period. In every run, three 3.2 mm punches were taken from each DBS card, generating 27 measurements over the three weeks for each of the seven pools. CK-MM concentrations in these punches were measured using the GSP Neonatal Creatine Kinase-MM kit (PerkinElmer, Waltham, MA, USA) with the GSP high-throughput analyzer as per the manufacturer’s instructions. The GSP instrument software was configured to output extrapolated results above the default upper reporting limit. Results were exported into spreadsheets by each laboratory and compiled at CDC, where they were graphed and analyzed for descriptive statistics using spreadsheet functions [18]. The kit controls were used by each laboratory to validate the results in every analytical run containing the prototype DBS-QC. No DBS-QC results were rejected; all were displayed graphically and included in calculating the descriptive statistics.

## 3. Results

### 3.1. Range, Variances, and Comparability between Laboratories

**Figure 1:**
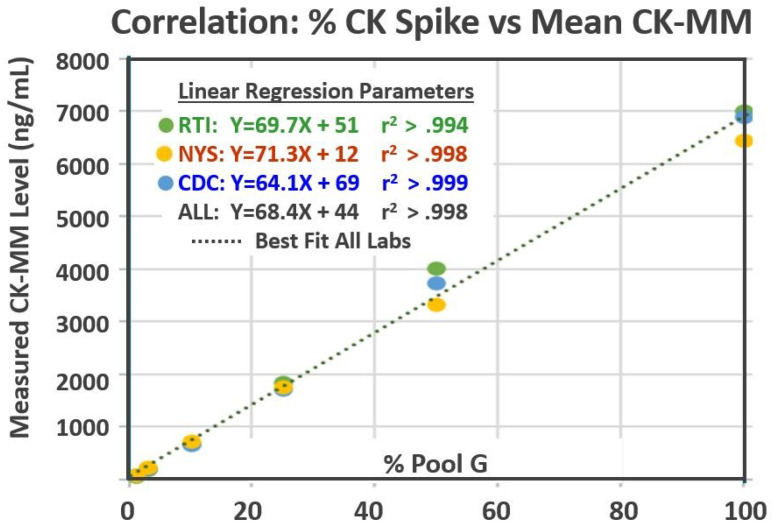
Correlation between the percentage of Pool G in Pools B–G and the mean CK-MM levels measured within each of the three laboratories.

**Figure 2:**
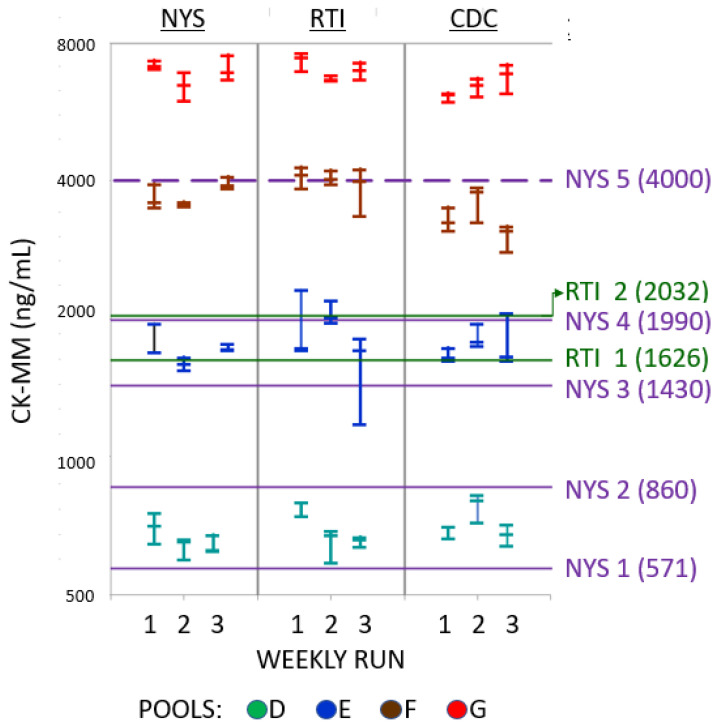
Distribution of CK-MM results from all laboratories for DBS made from Pools D–G. The three measured results from each laboratory for triplicate samples over the three weekly runs (X-axis) are displayed in the same column. Measured CK-MM levels are shown in logarithmic scale on the Y-axis. Cutoff values for screen positive results based on post-natal age are shown as horizontal lines labeled on the right, with CK-MM levels (in ng/mL) in parentheses. NYS actionable age-related cutoffs: NYS-1 > 168 h; NYS-2 72–167 h; NYS-3 48–71 h; NYS-4 < 47 h. All newborns with CK-MM results ≥4000 ng/mL (NYS-5) were immediately referred. Any newborn whose specimen was collected between 72 and 167 h was referred if the CK-MM was ≥860 ng/mL (NYS-2), and any newborn whose specimen was collected at ≥168 h was referred if the CK-MM was ≥571 ng/mL (NYS-1). The RTI-1 provisional cutoff was established with deidentified specimens from newborns less than 72 h old and applied to all screened newborns upon implementation. The provisional RTI-1 cutoff was then adjusted to RTI-2 as more data were acquired. All newborns with CK-MM above the cutoff that was active at the time were referred.

A total of 189 measured CK-MM results (63 from each laboratory) were collected over the three-week period (Table 2). The results for the kit controls were all within the acceptable ranges as determined by each laboratory. The measured CK-MM levels in the six spiked pools ranged from 58 ng/mL in Pool B to 7584 ng/mL in Pool G. Results from each laboratory were highly correlated with the relative proportion of CK-MM added to each of the spiked pools (Figure 1) [17]. There was no overlap in CK-MM results between any pools in any laboratory (see Table 2 and Figure 2). 

The mean values and variances for each pool were comparable among all three laboratories (Table 2). The difference between mean CK-MM levels from any two laboratories across all six spiked materials (Pools B–G) averaged 9.3%. The coefficient of variation (CV) for each spiked pool within each laboratory was 5–10% for 17 of the 18 data sets (Table 2). The overall CV of results for each spiked pool from all three laboratories was between 7% and 12%. 

### 3.2. Categorical Interpretation of CK-MM Results from All Pools

The 27 CK-MM results from each pool were evaluated based on both the multiple cutoffs used by NYS depending on post-natal age [7,8], and on a single preliminary provisional cutoff and a subsequent refined cutoff used by RTI for newborns less than 72 h old. By both NYS and RTI criteria, all CK-MM levels in Pools B and C measured in all three laboratories were screen-negative (that is, in the expected range for DMD-unaffected newborns). By the same criteria, all CK-MM levels in Pools F and G were screen-positive (that is, in an elevated range which would require further action such as repeat assay or referral). By NYS criteria, CK-MM levels in Pools D and E were distributed through a range that could require additional testing or follow-up depending on post-natal age [7,8], while all 27 of the CK-MM levels measured in Pool G would require immediate referral regardless of post-natal age. By RTI criteria, none of the 27 results on Pool D would require follow-up. Categorical results on Pool E differed depending on the use of the provisional or the refined cutoff: with the provisional cutoff, 22 of the 27 results would require follow-up; with the refined cutoff, only three would require follow-up.

## 4. Discussion

Renewed interest in population-based NBS for DMD is the result of technical advances in screening [5], the rapid development of new therapeutics [4], and the goal for a more rapid diagnosis after first signs appear, which currently averages more than 2 years [19]. This interest is reflected by several recent publications [4,5,6,7,8,9,10,11,12,13], all of which have used the same fluoroimmunometric assay specific for the CK-MM isoform [5]. The immunochemical specificity of this assay has been confirmed by its minimal cross-reactivity with the CK-MB and CK-BB isoforms [5]. Since CK-MM is a muscle isoform, the immunochemical specificity of this assay increases its clinical specificity as a biomarker for DMD. 

This report is the first multi-laboratory evaluation of the isoform-specific immunometric method of measuring CK-MM designed for NBS. Since it is limited to the use of contrived DBS, collaboration with NBS laboratories which were screening newborns in pilot studies was essential for evaluating the clinical relevance of the CK-MM levels included in the prototype DBS-QC set. The validity of the CK-MM distributions from the NYS and RTI studies is underscored by the similarity of their cutoff values for typical newborns (1990 and 2032 ng/mL, respectively), and by their mutual agreement with the provisional cutoff value (2040 ng/mL) provided in the CK-MM assay product insert (PerkinElmer GSP Neonatal Creatine Kinase-MM kit (3311-001U) instructions for use, version 1).

For NBS programs to establish and sustain DMD screening, laboratories will need reliable access to external QC materials to validate assays, conduct stability studies [20], ensure the consistency of test results, and meet regulatory requirements [15]. QC over a wide range is important, since CK-MM levels are variably elevated by birth trauma and then decline in the early post-natal period [21]. Based on results from the NYS pilot study [7,8], CK-MM levels in newborns can range from less than 100 ng/mL to as high as 19,000 ng/mL depending on post-natal age, birth trauma, and congenital disorders including DMD and other muscular dystrophies. 

In this study, the measured CK-MM values in Pools B–D encompassed the expected levels for typical newborns 24 to 72 h old, while CK-MM values in Pools F and G were in an elevated range. Results from an earlier multi-national evaluation of the same assay reported that CK-MM levels in the ranges commensurate with Pools F and G were found only in Duchenne-affected newborns [6]. Overall, the set of six spiked CK-MM DBS cards made from Pools B–G collectively spanned the expected ranges in typical newborns and the elevated ranges associated with higher risk for DMD reported in several recent studies (Table 3).

New York State used multiple cutoffs based on both newborn age and whether the elevation was Borderline (B), requiring re-test of a new specimen, or sufficiently elevated to require immediate Referral (R) for second-tier genetic testing and genetic counseling. RTI used a preliminary provisional cutoff and a subsequent refined cutoff for all newborns less than 72 h old. The other programs used a single cutoff for their follow-up actions, as described in their respective references. The Newborn Population column includes the age range of newborns at specimen collection as reported in each reference.

The amount of CK-MM used to spike Pool G was calculated to result in a concentration of 5000 ng/mL, based on the specific activity provided in the product insert. No information regarding CK-MM content measured immunometrically was provided, and no independent standard for accuracy of CK-MM measurements is currently available. The content of CK-MM measured immunometrically could also vary with respect to the method used to extract and purify the enzyme. These factors could potentially account for the 35% difference between the expected and measured CK-MM levels in Pool G. In the future, a workgroup formed by an authoritative standards agency may be able to establish a primary reference material to serve as a common calibrator.

Pre-analytical sources of variability are especially important with DBS specimens. The lack of homogeneous blood distribution throughout the spot, variations in the punched samples that are analyzed, and dissimilar accessibility of analytical reagents to the entire blood sample contained in the punch can all contribute to variability in the final result that is not related to the chemical analysis per se. Because the contrived DBS in this study were made from non-clotting blood applied uniformly using volumetric techniques, these issues are generally less problematic than they can be with heelstick samples collected from newborns. To ensure consistency, DBS materials distributed by NSQAP for use as external QC are assessed for homogeneity and stability using standard operating procedures approved under an accredited quality management system.

## 5. Conclusions

Based on the reference ranges established by the NYS and RTI DMD pilot studies as well as other recent published studies [4,6,7,9,10,11,12,13], we conclude that the set of CK-MM DBS evaluated in this study collectively spans the expected ranges in typical newborns and the elevated ranges associated with higher risk for DMD. The set therefore allows quality assessment over the wide range of CK-MM levels found in typical newborns, newborns affected by DMD, and newborns with moderately elevated CK-MM levels resulting from other causes, such as birth trauma, which then decline in the early post-natal period [8,21]. This evaluation of prototype DBS-QC materials demonstrates the feasibility of producing DBS to use as external controls for CK-MM assays used in newborn bloodspot screening.

## Figures and Tables

**Table 1:** Admixtures of Pool A (base pool) and Pool G (CK-MM spiked pool) in Pools B–F.

Pool	%Pool A	Pool G	Target CK-MM Conc (ng/mL)
A	100	0	0
B	99	1	50
C	97	3	150
D	90	10	500
E	75	25	1250
F	50	50	2500
G	0	100	5000

**Table 2:** Descriptive statistics from CK-MM measurements (ng/mL) over three weekly runs with results from each run in triplicate from all three laboratories.

	Pool	A	B	C	D	E	F	G
	**Mean**	3.51	62.4	185	689	1846	4007	6992
	**SD**	1.48	3.8	9	63	331	283	363
RTI	**CV (%)**	42	6	5	9	18	7	5
	**Min.**	1.36	58	174	588	1178	3362	6618
	**Max.**	5.33	68	199	795	2313	4285	7584
	**Mean**	5.07	68.2	201	661	1701	3730	6887
	**SD**	0.98	5.4	17	48	116	219	466
NYS	**CV (%)**	19	8	8	7	7	6	7
	**Min.**	3.62	62.1	177	597	1541	3494	5984
	**Max.**	6.08	80.7	233	755	1954	4087	7537
	**Mean**	3.94	79	220	717	1757	3319	6432
	**SD**	2.17	5.4	19	61	153	343	404
CDC	**CV (%)**	55	7	8	9	9	10	6
	**Min.**	1.07	70.8	189	640	1618	2800	5957
	**Max.**	6.91	89.3	248	825	2053	3863	7181
	**Mean**	4.17	69.9	202	689	1768	3685	6771
	**SD**	1.32	4.2	12	61	285	330	369
All labs	**CV (%)**	41	12	10	9	12	11	7
	**Min.**	1.07	58	174	588	1178	2800	5957
	**Max.**	6.91	89.3	248	825	2313	4285	7584

**Table 3:** CK-MM cut-off levels for actionable follow-up published by different NBS laboratories using the same fluoroimmunoassay.

NBS Program	Newborn Population	Cutoff (ng/mL)	Reference
New York State Department of Health	≥168 h 72–167 h 48–71 h ≤47 h	571 (R) 571 (B) 860 (R) 1430 (B) 4000 (R) 1990 (B) 4000 (R)	[8] Park et al. 2022
Early Check Program, RTI International	≤72 h (Provisional) ≤72 h (Refined)	1626 2032	[9] Kucera 2021
Supplemental Duchenne Muscular Dystrophy Newborn Screening	Males 24–48 h Females 24–48 h	1080 958	[4] Parad et al. 2021
California/Denmark Population Study	US 12–60 h Danish ≥ 48 h	1190 675	[6] Timonen et al. 2018
National Taiwan University Hospital	All Newborns (Mean Age 2 Days)	750	[13] Chien et al. 2022
Zhejiang Province China	Males 3–5 days	700	[10] Ke et al. 2017
Guangzhou City China	Males and Females 48 h to 7 days	800	[11] Jia et al. 2022
Henan Province China	Males 48–72 h	472	[12] Jia et al. 2023

## Data Availability

The data presented in this study are available on request from the corresponding author.
